# Marine Microalgae, *Spirulina maxima*-Derived Modified Pectin and Modified Pectin Nanoparticles Modulate the Gut Microbiota and Trigger Immune Responses in Mice

**DOI:** 10.3390/md18030175

**Published:** 2020-03-21

**Authors:** H.P.S.U. Chandrarathna, T.D. Liyanage, S.L. Edirisinghe, S.H.S. Dananjaya, E.H.T. Thulshan, Chamilani Nikapitiya, Chulhong Oh, Do-Hyung Kang, Mahanama De Zoysa

**Affiliations:** 1 College of Veterinary Medicine and Research Institute of Veterinary Medicine, Chungnam National University, Yuseong-gu, Daejeon, 34134, Korea; surathsanda@gmail.com (H.P.S.U.C.); thilomaliyanage@gmail.com (T.D.L.); shan.lakmal09011@gmail.com (S.L.E.); shsdananjaya@gmail.com (S.H.S.D.); thimira.thulshan.jayathilaka@gmail.com (E.H.T.T.); chamilani14@gmail.com (C.N.); 2 Jeju Marine Research Center, Korea Institute of Ocean Science and Technology (KIOST), Jeju 63349, Korea; och0101@kiost.ac.kr; 3 Department of Ocean Science, University of Science and Technology (UST), Jeju 63349, Korea

**Keywords:** Bacteroidetes, Firmicutes, intestinal alkaline phosphatase, metagenomics, mucin, modified SmP, *Spirulina maxima*

## Abstract

This study evaluated the modulation of gut microbiota, immune responses, and gut morphometry in C57BL/6 mice, upon oral administration of *S. maxima*-derived modified pectin (SmP, 7.5 mg/mL) and pectin nanoparticles (SmPNPs; 7.5 mg/mL). Metagenomics analysis was conducted using fecal samples, and mice duodenum and jejunum were used for analyzing the immune response and gut morphometry, respectively. The results of metagenomics analysis revealed that the abundance of Bacteroidetes in the gut increased in response to both modified SmP and SmPNPs (75%) as compared with that in the control group (66%), while that of Firmicutes decreased in (20%) as compared with that in the control group (30%). The mRNA levels of mucin, antimicrobial peptide, and antiviral and gut permeability-related genes in the duodenum were significantly (*p* < 0.05) upregulated (> 2-fold) upon modified SmP and SmPNPs feeding. Protein level of intestinal alkaline phosphatase was increased (1.9-fold) in the duodenum of modified SmPNPs feeding, evidenced by significantly increased goblet cell density (0.5 ± 0.03 cells/1000 µm^2^) and villi height (352 ± 10 µm). Our results suggest that both modified SmP and SmPNPs have the potential to modulate gut microbial community, enhance the expression of immune related genes, and improve gut morphology.

## 1. Introduction

Prebiotics such as fructooligosaccharides (FOS) and inulin are known to selectively stimulate beneficial intestinal microbiota which convert these prebiotics into bioactive metabolites. Most prebiotics, a form of soluble dietary fiber, which are complex polymers present in plant-based foods, are well-known for their beneficial fermentable products such as short chain fatty acids (SCFAs) and immunomodulatory properties [1]. For instance, dogs fed with a fermentable plant-based fiber diet showed significant alterations in the proportion of T cells in gut-associated lymphoid tissue and their in vitro response to mitogens [2]. The effect of dietary fiber intake on gut microbiota has been described with different kinds of fiber such as inulin, oats β-glucan, cellulose, etc. Several symbiotic gut microbiota are closely associated with a broad range of beneficial functions, including the biosynthesis of essential nutrients, digestion of dietary components, enhancement of digestive enzyme functions, and immunity protection against enteric pathogens [3]. Conversely, a disrupted and altered gut microbiota is correlated with several diseases and health complications [4].

Pectin is an anionic and amorphous complex polysaccharide composed of side chains (e.g., xylose, arabinose, glucose) linked to galacturonic acid backbone [5] and is classified as a soluble dietary fiber. In the food industry, pectin has been widely used as a thickening or gelling agent; however, due to its specific bioactivities against human diseases (e.g., obesity and cancer), it is gaining popularity in the pharmaceutical field [6]. Although apple pomace and lemon peel have been mostly used as commercial pectin sources, scientists have become interested in finding novel sources for pectic polysaccharide isolation. However, the chemical composition of pectin heavily depends on its source and is affected by the isolation and purification techniques used [6].

The *Spirulina* (*Arthrospira*) species (i.e., *S. platensis* and *S. maxima*) have attracted attention as a dietary supplement due to its nutritional value and potential health advantages (e.g., anti-obesity and immune modulatory effects) [7]. The *S. maxima* extract treatment has reduced weight gain and the index of white adipose tissues, as well as induced AMP-activated protein kinase (AMPK) pathway and sirtuin 1 (SIRT1) to suppress the development of pathophysiological mechanisms associated with obesity in high-fat diet-induced obese rats [8]. Consumption of Immulina, a polysaccharide isolated from *Arthrospira platensis*, significantly induced Th1 cytokines (TNF-α, IL-2, and IFN-γ) in healthy males and stabilized increased IL-2 levels for 56 days [9]. Furthermore, *Spirulina*-derived C-phycocyanin, increased the secretion of inflammatory cytokines TNF-α, IL-1β, and IL-6 in murine macrophages [10]. In our previous study, we isolated the pectin from *S. maxima* (SmP) and described the immune modulation in zebrafish [11]. Moreover, preliminary experimental results indicate the potential prebiotic and wound healing effects of SmP in zebrafish model (unpublished data). However, there is no available information on prebiotic effects of pectic polysaccharides isolated from *S. maxima* on gut microbiota, immunomoduation, and gut morphometry changes in mice. Therefore, we hypothesized that modified SmP and SmPNPs as a lead marine biomaterial with multiple functions which could improve the animal health.

This study aimed to investigate the effects of modified *S. maxima* pectin (SmP) and pectin nanoparticles (SmPNPs) on the modulation of mice gut microbiota and immune responses, including antimicrobial, antiviral, and inflammatory cytokines. Moreover, gut histomorphometry changes were analyzed upon the supplementation of modified SmP and SmPNPs in a mouse model.

## 2. Results

### 2.1. Growth Performance and Blood Glucose Levels of Mice upon Modified SmP and SmPNPs Supplementations 

**Figure 1:**
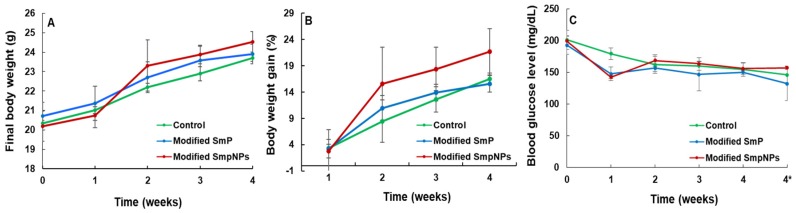
Growth performance and blood glucose level of mice during 4 weeks of modified SmP and SmPNPs treatment. (**A**) Body weight (g); (**B**) Body weight gain percentage (%); (**C**) Blood glucose levels (mg/dL). 4* denoted the fasting glucose level at the end of the experiment (4th week); (means ± standard deviation, n = 4 per replicate, 3 replicates per group).

Final bodyweight was not significantly different (*p* > 0.05) among control and modified pectin groups (Table 1 and Figure 1A). Although weight gain (g), weight gain percentage (%), and specific growth rate percentage (SGR %) were not significantly different, the modified SmPNPs treated group (Table 1 and Figure 1B) showed the highest weight gain % (21.65) and SGR % (0.70) levels, while the modified SmP treated group showed the lowest weight gain % (15.54) and SGR % (0.51) levels compared with the control (16.52% and 0.55%, respectively). The water intake per mouse per day was slightly higher in both groups supplemented with modified SmP (4.33 mL) and SmPNPs (4.34 mL), as compared with the control group (4.05 mL). The total modified SmP and SmPNPs intake were almost identical (45.36 and 45.64 g/mouse/4 weeks, respectively). The blood glucose level was consistent throughout the study period with slight fluctuations in the modified SmP (158.72 ± 20.0 mg/dL), SmPNPs (165.82 ± 21.0 mg/dL), and the control group (171.25 ± 19.00 mg/dL). Furthermore, there was no significant difference in weekly and fasting (fourth week) blood glucose levels in both treatment groups (Table 1 and Figure 1C).

### 2.2. Effects of Modified SmP and SmPNPs Supplementations on the Mouse Gut Microbiota

#### 2.2.1. Metagenomic Sequencing and Diversity Analysis 

**Figure 2:**
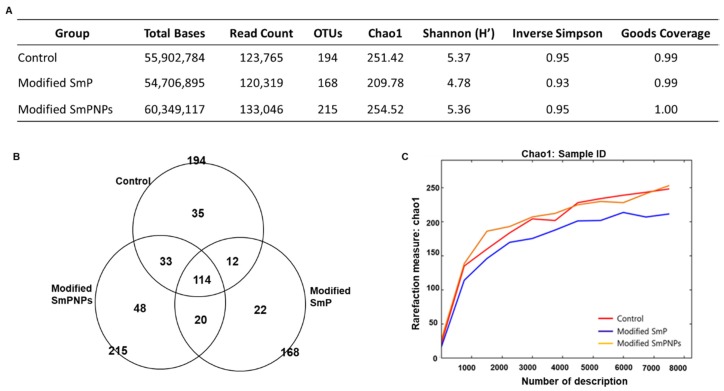
Metagenomics analysis of mouse fecal matter. (**A**) Sequencing reads, Operational Taxonomic Units (OTUs) count and diversity indices; (**B**) Venn diagram of observed OTUs; (**C**) Rarefaction curves of control, modified SmP and SmPNPs treated mice (n = 4 per replicate, 3 replicates per each group).

To evaluate the effects of modified SmP and SmPNPs supplementations on the gut microbial community, we analyzed the fecal microbial composition by 16S rRNA metagenomics sequencing. The highest read count (133,046) and number of Operational Taxonomic Units (OTUs) (215) were reported in the modified SmPNPs group. The modified SmP group showed a lower read count (120,319) and OTUs (168) than the control group. The alpha diversity of gut microbiota was higher in modified SmPNPs and control groups than the group fed with modified SmP. Although the diversity values were not significantly higher or lower, both the Shannon–Weiner and Simpson’s diversity indices were similarly higher in the modified SmPNPs (5.36 and 0.95, respectively) and the control (5.37 and 0.95, respectively) groups regardless of OTU count, than the modified SmP group (4.78 and 0.93, respectively). The highest Chao1 index and Good’s coverage indices were also reported in the modified SmPNPs group (Figure 2A). As illustrated in the Venn diagram, 114 OTUs were identified as core OTUs, which were common to all three groups. The modified SmPNPs group showed the highest unique OTUs (48) as compared with the modified SmP (22) and the control (35) groups (Figure 2B). Rarefaction curves that displaying species richness also have become more plateaued to the right in the SmPNPs treated mice (Figure 2C), further confirming the high richness in the microbial community. This indicates the saturation of sequencing reads of the samples, and we decided further sequencing would not be necessary for searching more OTUs.

#### 2.2.2. Taxonomic Analysis 

**Figure 3:**
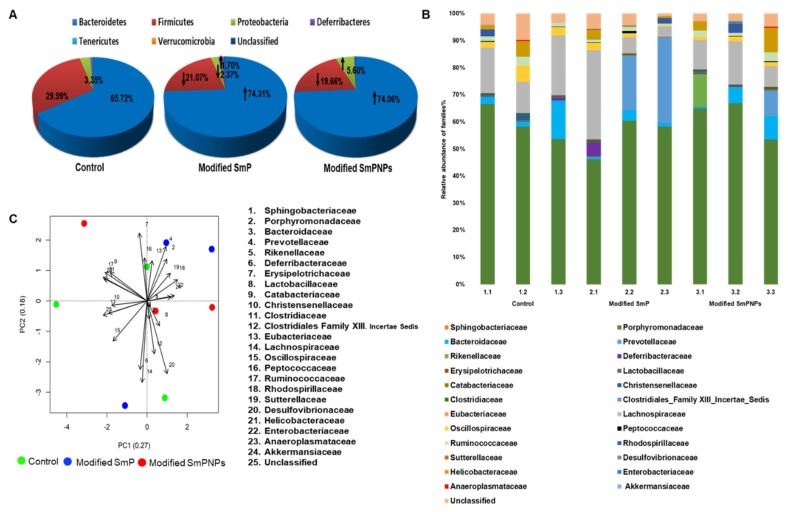
Diet-specific changes of taxonomic composition of fecal microbial community of control, modified SmP, and modified SmPNPs treated mice. (**A**) Comparison of relative abundance of metagenomics based gut microbial phyla; (**B**) Comparison of relative abundance of metagenomics based gut microbial families; (**C**) Principal component analysis (PCA) of relative abundance of gut microbiota families. The percentage variance of PC1 and PC2 are represented in x and y axis, respectively, (Software version: R 3.6.1, packages. Vegan). (n = 4 per replicate, 3 replicates per each group).

According to the taxonomic analyses, the increase of Bacteroidetes was notable in the pectin supplemented group as compared with the control group. At the phylum level, higher relative abundances of Bacteroidetes (74.31%) and lower levels of Firmicutes (21.07%) were observed in the modified SmP group as compared with the control group; however, these values were not significant (*p* > 0.05). Interestingly, supplementation with the modified SmPNPs significantly increased (*p* < 0.05) Bacteroidetes levels (74.06%) and lowered Firmicutes levels (19.66%) as compared with the control group (65.72% and 29.99%, respectively). Moreover, modified SmPNPs (Figure 3A) showed a high percentage of Proteobacteria (5.60%), while modified SmP showed a high abundance of Deferribacteres (1.70%). At the class level, the highest abundant classes of all three groups were Bacteroidia, Clostridia, and Epsilonproteobacteria. From these, the relative abundance of class Bacteroidia was significantly higher (74%, *p* < 0.05), and class Clostridia was significantly lower (19%, *p* < 0.05) in the group fed with modified SmPNPs vs. the control group (65% and 29%, respectively). This difference was also observed in the order level of the group fed with modified SmPNPs, with respect to the relative abundance of Bacteroidales and Clostridiales as compared with the control group (Supplementary Figure S1). The microbial community family distribution of the three groups was distinctively different (Figure 3B). In the phylum Bacteroidetes, the *Porphyromonadaceae* family was found to be the highest abundant; its relative abundance was almost similar in the control (60%) and modified SmPNPs (61.6%) groups and slightly lower in the modified SmP (55.4%) group. In the modified SmP group, the next highly abundant families in the phylum Bacteroidetes were *Prevotellaceae* (18.6%), *Bacteroidaceace* (1.9%), and *Rikenellaceae* (0.04%). This order was slightly different in the modified SmPNPs group as *Bacteroidaceace* (5.1%), *Rikenellaceae* (4.2%), and *Prevotellaceae* (3.6%), respectively. In the control group, an additional family found belonging to the phylum Bacteroidetes was *Bacteriodaceae* (6.1%). With respect to the phylum Firmicutes, the most abundant family percentage was *Lachnospiraceae* in all groups; the following order was observed: control (16.4%), modified SmP (13.1%), and modified SmPNPs (10.9%). The control group showed a higher abundance percentage in other *Firmicutes* families, such as *Ruminococcaceae* (7.1%) and *Oscillospiraceae* (3.8%), which were considerably lower in the groups treated with both modified SmP (3.5% and 1.5%, respectively) and modified SmPNPs (4.6% and 1.5%, respectively).

Considering principal component analysis (PCA) of relative abundance of gut microbiota families, the first two principal components collectively displayed ~45% of total variance; only two treatment replicates of each treatment groups (modified SmP and modified SmPNPs) appeared to be clustered closely (Figure 3C), whereas relative scattering was observed among the control replicates. This indicates a trend of gut microbial modulation with the modified SmP and modified SmPNPs supplementations. However, permutational multivariate analysis of variance (PERMANOVA) values of the relative abundance of gut microbial families were not significantly different (*p* > 0.05) in modified SmP and modified SmPNPs supplemented groups as compared with the control group (Supplementary Figure S2).

### 2.3. Modified SmP and SmPNPs Supplementations Displayed Immunostimulation in Mice 

**Figure 4:**
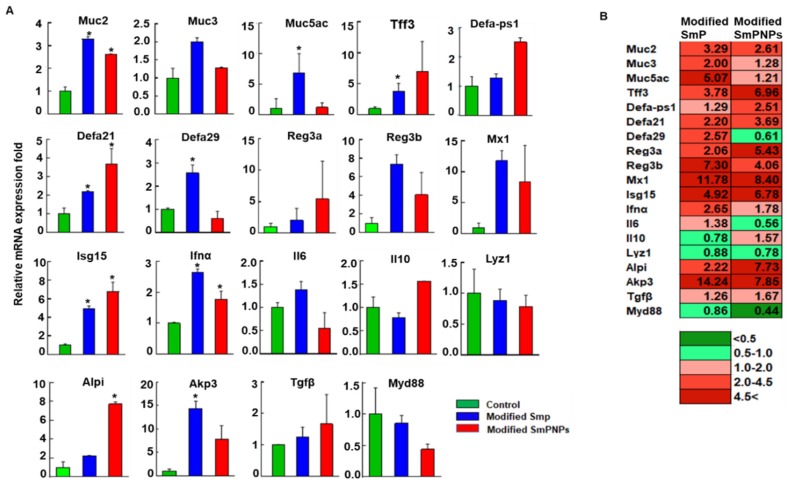
Transcriptional responses of immune related genes in modified SmP and SmPNPs supplemented mice. (**A**) Relative mRNA expression in control, modified SmP, and modified SmPNPs treatment groups. Relative expression-fold was presented as mean ± standard error. Asterisk mark is used to indicate the significant difference between the pectin treatments and control (3 replicates/group); (**B**) Comparison of relative expression fold between the treatment groups by color schematic representation. Basal expression level was considered as 1.0-fold; upregulated and down regulated expression were considered as >1.0- and <1.0-folds, respectively.

We analyzed the expression of selected immune response genes in the duodenum to understand the effect of dietary pectin. Relevant main functions of the selected genes are included in Supplementary Table S2. On the one hand, in the modified SmP supplemented mice, mucin (Muc2, and Muc5ac), antimicrobial peptides (Tff3, Defa21, Defa29, and Reg3b), antiviral genes (Ifnα, Isg15, and Mx1), and gut permeability related alkaline phosphatase 3 (Akp3) were significantly upregulated (*p* < 0.05) over 2-fold as compared with the control group. Similarly, Muc3, Reg3a, and Alpi were upregulated (>2-fold), but it was not significant in the modified SmP group. In addition, the fold values of Defa-ps1, Il6, and Tgfβ genes (Figure 4A) remained at a basal level (1.0- to 1.5-fold) while fold values of Il10, Lyz1, and Myd88 were slightly suppressed (<1.0-fold), yet the fold change was not significant in the modified SmP group. On the other hand, in the modified SmPNPs supplemented group, over 2-fold significant upregulation (*p* < 0.05) was observed in Muc2 (2.61-fold), Defa21 (3.69-fold), Isg15 (6.78-fold), and Alpi (7.73-fold) genes. The Mx1 (8.40-fold), Tff3 (6.96-fold), Defa-ps1(2.51-fold), Reg3a (5.43-fold), Reg3b (4.06-fold), Akp3 (7.85-fold) genes also showed over 2-fold upregulation, although not significant as compared with the control group. Furthermore, Muc3 (1.28-fold), Muc5ac (1.21-fold), Ifnα (1.78-fold), Il10 (1.57-fold), and Tgfβ (1.67-fold) genes were slightly elevated or remained at a basal level; however, Defa29 (0.61-fold), Il6 (0.56-fold), Lyz1 (0.78-fold), and MyD88 (0.44-fold) genes were found to be slightly suppressed (<1.0-fold) as compared with the control group (Figure 4A,B). With respect to the both modified SmP and modified SmPNPs treated groups, Muc2, Tff3, Defa21, Reg3a/b, Isg15, Mx1, Alpi, and Akp3 genes were upregulated over 2-fold, while Lyz1 and Myd88 were found slightly suppressed.

### 2.4. Intestine Alkaline Phosphatase (IAP) Expression by Immunoblotting Analysis

**Figure 5:**
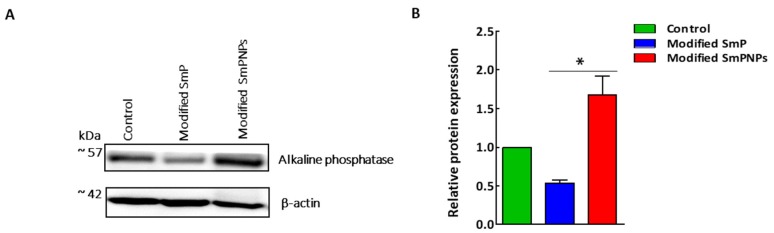
Immunoblot analysis of duodenum intestine alkaline phosphatase expression (IAP) of mice fed with modified SmP and SmPNPs supplemented diet and the control. (**A**) IAP expression. β-actin was used to confirm equal loading of proteins; (**B**) Quantitative analyses of IAP expression in modified SmP and SmPNPs, which normalized to β-actin and compared with the control. Data are expressed as the mean ± standard error (n = 3).

The relative IAP expression in modified SmP showed downregulation (0.57-fold) as compared with the control sample, while modified SmPNPs showed upregulation (1.92-fold) as compared with the control group (Figure 5B). Duodenum samples showed different level of IAP expression, while almost equal expression levels of β-actin among three samples (Figure 5A).

### 2.5. Histological Analysis

**Figure 6:**
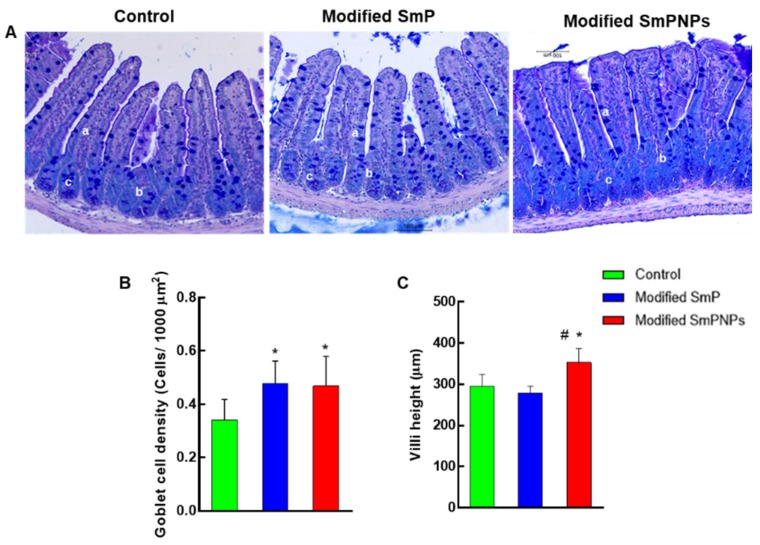
Histological analysis of jejunum morphometry of mice fed with modified SmP and SmPNPs supplemented diet as compared with control. (**A**) Light micrographs of Alcian blue and periodic acid=Schiff (AB-PAS) stained histological sections; (**B**) Comparison of goblet cell density; (**C**) Comparison of villi height in control, modified SmP, and modified SmPNPs treated mouse gut. a, villus goblet cells; b, crypt goblet cells; c, paneth cells. * Significantly (*p* < 0.05) higher goblet cell density and villi height as compared with the control; # significantly (*p* < 0.05) higher villi height in modified SmPNPs supplemented group as compared with modified SmP group.

The effect of modified SmP and SmPNPs supplementations to mice was compared with the control group on intestinal morphology and morphometry using the histological analysis of gut (jejunum). Light micrographs of histological sections from all three groups did not show any pathological changes such as necrosis and inflammation (Figure 6A). The alcian blue (AB) and periodic acid-Schiff (PAS) stained sections showed a significant (*p* < 0.05) increase of goblet cell density (0.5 ± 0.03 cells/1000 µm^2^) in modified SmP and SmPNPs supplemented mice as compared with the control group (0.3 ± 0.02 cells/1000 µm^2^) (Figure 6B). Furthermore, villi height in modified SmPNPs (352 ± 10 µm) supplemented group was significantly increased as compared with both the control (295 ± 8 µm) and modified SmP (279 ± 4 µm) groups (Figure 6C).

## 3. Discussion

Pectin, a complex polysaccharide which is present in higher plant cell walls, can also be found in gymnosperms, pteridophytes, and bryophytes. Relatively few studies have been carried out to characterize pectin in algal species and pectic polysaccharides in charophytes, which is the closest evolutionary branch of land plants [12]. An exopolysaccharide containing 83% galacturonic acid was found to be synthesized by *Microcystis flosaquae;* however, there is currently no available information of its structural resemblance to pectin [13]. Thus, in this study, we isolated pectic polysaccharide from *S. maxima*, which is found to have a close resemblance to pectin. Furthermore, the use of *S. maxima* as a raw material can be advantageous due to its already established process for large scale production (7).

Modifications to pectin can be done enzymatically and non-enzymatically. In the latter, modifications to pectin such as depolymerization and demethoxylation have been acquired through pH, temperature, or pressure changes [14]. We created modified SmP by applying high temperature and pressure conditions to pectic polysaccharide isolated from *S. maxima*, which led to decreased rates of β-elimination of pectin, but increased rates of demethoxylation [15]. In addition, we obtained the nano derivative form of SmP via sonication, in order to reduce the particle size of modified SmP molecules mechanically rather than using any chemical methods, so the whole effect is from the pectin. Pectin and modified pectin isolated from other sources have displayed different bioactivities in mammalian models [16]. In our study, increased water consumption with both modified SmP and SmPNPs supplementations suggests that the mice drink more water under pectin treatment, which could be beneficial in maintaining hydration. In accordance with our results, it has been reported that, dietary pectin supplementation resulted in an increased food and water consumption, without weight gain in mice fed with a high cholesterol diet [17]. The apple pectin also decreased weight gain in mice fed with high fat diet [18]. However, the modified SmPNPs treatment increased the weight gain. We assumed that the smaller particle size of modified SmPNPs could have increased digestibility and availability of nutrients.

Glucose homeostasis in mice was not affected by supplementation of modified SmP or SmPNPs. Pectin interaction with the blood glucose level has produced controversial results in previous studies. For example, in Chen et al., pectin supplementation in mice fed a high cholesterol diet did not affect fasting blood glucose level or glucose tolerance [17]. Nelson et al. described that soluble fiber diet containing pectin lowered post-cardinal plasma glucose concentrations in dogs [19].

Analysis of gut microbiota can be done using fecal matter, mucosal biopsy, or luminal continents. Fecal samples are remained more preferable, since their collection is easier than biopsy samples [20]. In the fecal microbiota analysis results, lower read counts and OTUs were detected in the SmP group as compared with the control group, signifying that modified SmP treatment could have lowered some of the microbiota in the gut. However, the highest read count and OTUs in the modified SmPNPs administrated group indicated that nanoscale pectin could have agreater effect probably by providing more surface area and simpler fragments as compared with original pectin molecules. Furthermore, higher Shannon and Simpson’s diversity indices were observed in the control and both pectin treated groups, indicating the species richness and evenness among taxa in all groups (Figure 2A). Despite not being statistically significant, mice supplemented with modified SmP showed slightly lower species richness. The higher microbial richness of modified SmPNPs treated mice is related to the smaller particle size of the product, which provides a higher surface area for microbial growth than the original longer molecules of modified SmP. The increase of Bacteroidetes seen in pectin-supplemented mice is consistent with previous studies such as the increased proportionate abundance of Bacteroidetes of cecal microbial composition resulting from supplementation with an apple pectin diet [21]. This trend has also been observed in human fecal microbiota as a result of apple pectin fermentation [21]. In contrast, decreased level of Firmicutes has been found to reduce high-fat diet obesity [21]. In our study, reduction of Firmicutes was mainly due to the reduction of the class clostridia species. Unlike Bacteroidetes, most Firmicutes are unable to degrade pectin except a few such as *Eubacterium eligens*, which is known to be strongly promoted by pectin [22]. A notable link between the Firmicutes/ Bacteroidetes ratio and obesity was assessed in a study where Firmicute species were dominant in obese individuals as compared with lean ones [23]. Furthermore, fiber enrichment with citrus and apple pectin has resulted in increased Bacteroidetes in cecal contents and reduced weight gain [21,24]. Among rumen bacteria, many strains belonging to genus *Prevotella* have been found to encode pectin methyl esterases which is an evidence that they are predominantly utilizing pectin [25]. The higher levels of *Prevotella* species in modified SmP and SmPNPs treatment groups of in this study could also be associated with pectin degradation through carbohydrate-active enzymes. Interestingly, we discovered *Prevotella loescheii* was present in the fecal samples of modified SmPNPs fed mice. *P. loescheii* is a strictly anaerobic bacterium in the gut, and has been identified as an opportunistic pathogen, but such incidences are rare [26]. Table 2 indicates the some of the notable changes in species level and their modulatory effects.

Taken together, our results showed that the intestinal microbiome was distinctively affected by the supplementation of modified SmP and SmPNPs, yet statistical analysis of collective effects on the different microbial families present in the gut by our treatments were shown as not significant. We assume that an extended duration of feeding is needed to understand the long-term significant pectin effect on intestinal microbiota.

Pectin’s immunomodulatory properties which includes both activation and suppression of target genes are mostly determined by its chemical structure. For example, a comparison of immunomodulatory activity of different pectin types revealed that pectin which contains less than 75% galacturonic acid residues can enhance the positive immune responses (33). In our study, modified SmP and SmPNPs supplementations in mice induced the expression of wide array of immune response genes. Therefore, studies with further chemical and structural characterization are needed for a better understanding of the immunomodulatory function of Spirulina-derived modified SmP. Soluble fiber such as pectin increases mucin (e.g., mucin2, mucin3, and mucin5ac) secretion in the small intestine with or without increasing intestinal goblet cell number [33]. *Tff3* is also mainly expressed with *Muc2* in goblet cells and plays a major role in mucosal surface regeneration and repair [34,35]. The *α-defensins* and *Reg3a*/*b* are produced by Paneth cells, which are important in innate enteric immunity and are also known to regulate based on changes in intestinal microbiome composition [36,37]. Induction of IAP in the small intestine was present in obese mice supplemented with a high-fat, apple pectin diet [20]. Moreover, *Ifn α*, *Isg 15*, and Mx1 genes, which are activated upon microbial infections such as viral invasions, play a critical role in innate immunity [38]. In our study, these genes were notably upregulated and revealed that modified SmP and SmPNPs can exert a protective function in mice duodenum through their immunostimulatory property.

Intestinal alkaline phosphatases (IAPs) are important for gut immunity and protection, affecting gut physiology by maintaining the surface pH in the duodenum and modulating bacterial LPS-induced inflammation through detoxifying LPS [39,40]. Furthermore, IAP has been reported to act as an anti-inflammatory factor by inhibiting both Toll like receptor 4 (Tlr4) and myeloid differentiation primary response gene 88 (Myd88) dependent inflammatory cascades [41]. Our protein expression result showed the elevated IAP expression level in the modified SmPNPs group (Figure 5) which further validated the marked upregulation of Alpi and Akp3 mRNA expression in the modified SmPNPs group (Figure 4). The slight downregulation of Myd88 was possibly due to the direct action of modified SmP and SmPNPs or the indirect action of an increased level of IAP, supporting the anti-inflammatory action upon feeding of modified SmP and SmPNPs.

The modified SmP and SmPNPs have shown improved gut morphometry by increasing the density of goblet cells per villi and villi height (Figure 6). *Muc2,* produced by goblet cells, is important for gut barrier function as it forms thick inner layers over the gut epithelium helping block the access of pathogenic microbes to the gut epithelium [30]. Gut morphometry responses were diverse among the different fiber diets such as pectin, cellulose, oat β-glucan, or inulin. For example, supplementation with low methoxyl pectin increased the number of goblet cells in small intestine in rats [34], and carboxyl methyl cellulose was reported to increase crypt depth and serosa thickness in chickens [41]. Therefore, our results suggest that the improvements in gut morphology by modified SmP and SmPNPs treatments strengthen the health benefits of *Spirulina*-derived pectin.

In conclusion, this study provides a novel insight into the positive impacts of modified *Spirulina*-derived pectin and its nano-derivatives on gut microbial community through a beneficial shift in gut microbiota. This is relatively concurred with the effects of apple or citrus originated pectin which are one of the most abundant forms of pectin. Induction of immune-related gene responses and improvement of gut morphometry further support the potential of immunomodulation activity of the modified SmP and SmPNPs supplementations through oral routes. Thus, the current study suggests the prebiotic potential of *Spirulina*-derived modified pectin and encourages further studies to understand the underlying mechanisms of immunomodulatory properties.

## 4. Materials and Methods

### 4.1. Preparation and Characterization of Modified SmP and Modified SmPNPs 

SmP was isolated from a cyanobacterium, *S. maxima*, and it was modified by providing high temperature and pressure conditions over a period of predetermined time to make modified SmP. Modified SmPNPs were prepared by sonication of modified SmP (Sonics & Materials. Inc. Newtown, CT, USA). Briefly, modified SmP was dissolved in deionized water and sonicated under amplitude 30%, 10:10 sec pulse at 4 °C for 30 min. Sonicated pectin solution was centrifuged at 3500 rpm for 15 min. The collected supernatant (modified SmPNPs) and modified SmP were used for particle size distribution and zeta potential analysis by Zetasizer S-90 Malvern instruments (Malvern, UK). Modified SmP and SmPNPs had an average particle size of 152.90 and 64.11 nm, respectively, and zeta potential of −24.4 and −24.6 mV, respectively (Supplementary Table S1). These two samples were used for our experimental feeding trials.

### 4.2. Animals and Experimental Design 

The C57BL/6 mice (male), with an initial bodyweight of 20.41 ± 0.55 g, were purchased from Orient Bio Inc. (Seongnam, Republic of Korea). The mice were maintained in the housing facility under pathogen-free conditions in a controlled environment (12 h light/dark cycle, 22 ± 2 °C, relative humidity-50 ± 5%) with free access to standard pellet food and sterilized water. After the acclimatization (7 days), animals were divided into 3 groups as control, modified SmP, and SmPNPs (triplicates, n = 4). Modified SmP and SmPNPs were administrated with drinking water for 4 weeks at a dose of 7.5 mg/mL (1.62 ± 0.01g/kg body weight/day). The water intake (daily) and the body weight (weekly) of the mice were measured. Weight gain % and SGR % were determined according to, weight gain % = ((Wf − Wi)/ Wi) *100 and SGR % = ((ln Wf − ln Wi)/t) *100, Where, Wi, Wf, and t denote initial body weight, final body weight, and study period (days), respectively. The blood glucose level (mg/dL) was determined using a glucometer (Accu-Chek, Roche, Germany) using the tail tip amputation method [42]. The experiment protocols were approved by the Animal Experimental Ethics Committee (CNU-01105) of the Chungnam National University, Daejeon, Republic of Korea.

### 4.3. Sample Collection, Genomic DNA Extraction, and Library Construction

For metagenomics analyses, fecal samples were collected and snap-frozen in liquid nitrogen at the end of four weeks. Then, the duodenum was surgically removed, snap-frozen, and stored at −80 °C prior to RNA isolation and immunoblot analysis. For histomorphological studies, the jejunum was collected, washed, and fixed in 10% neutral buffered formalin. Genomic DNA was extracted from fecal samples using QIAamp DNA Stool Mini Kit (QIAGEN GmbH, Hilden, Germany) according to the manufacturer’s instructions. PCR amplification was performed using primers targeting the V3 to V4 regions (F: 5′-GAGTTTGATCMTGGCTCAG-3′) and R: (5′-WTTACCGCGGCTGCTGG-3′) of the 16S rRNA gene with extracted DNA. Secondary amplification for attaching the Illumina NexTera barcode was performed with an i5 and i7 adaptors.

### 4.4. Next Generation Sequencing (NGS) and Metagenomics Analysis 

Quality and product size were assessed on a Bioanalyzer 2100 (Agilent Technologies Inc., Santa Clara, CA, USA) using a DNA 7500 chip. Amplicons were pooled and the sequencing was performed with the Illumina MiSeq Sequencing system (Illumna Inc., San Diego, CA, USA) according to the manufacturer’s instructions. Processing raw reads began with a quality check and filtering of low quality (<Q25) reads by Trimmomatic 0.321. After QC pass, paired-end sequence data were merged together using PandaSeq2. Then, primer sequences were trimmed with an in-house program at a similarity cutoff value of 0.8. The EzTaxon database was used for taxonomic assignment using BLAST 2.2.224, and pairwise alignment5 was used to calculate % similarity. Uchime6 and the non-chimeric 16S rRNA database from EzTaxon were used to detect chimera on reads that contained a less than 97% best hit similarity rate. Finally, sequence data were clustered using CD-HIT7 [43] and UCLUST8 [44], and alpha diversity indices (Shannon–Weiner, Simpson’s, and Chao1 indices) were determined.

### 4.5. RNA Extraction and qRT-PCR Analysis 

The total RNA was extracted from the duodenum using Tri Reagent™ (TaKaRa Bio Inc., Shiga, Japan) following standard protocol. The quality and concentration were determined, and the first strand cDNA was synthesized using 2.5 μg of total RNA by PrimeScript™ cDNA kit (TaKaRa, Bio Inc., Shiga, Japan). Expression of selected immune response genes was performed by qRT-PCR (Thermal Cycler Dice Real Time System, TaKaRa Bio Inc., Shiga, Japan). Reaction mixture (14 μL) included 5 μL of cDNA as a template, 7 μL of THUNDERBRID^®^ SYBR^®^ qPCR mix (Toyobo Co., Ltd., Osaka, Japan) and 1 μL of gene specific forward and reverse primers (10 pmol/μL). Relevant primer sequences are included in Supplementary Table S2. Data were normalized using GAPDH (housekeeping gene) and expression fold was calculated by 2^−ΔΔCT^ method [45].

### 4.6. Immunoblot Analysis for IAP Expression 

Duodenum tissue samples were homogenized in lysis buffer, pH 7.6 (ProEX^TM^ CETi, Trans Lab, Inc, Daejeon, Republic of Korea) containing non-ionic detergent, protease inhibitors, and phosphatase inhibitors. The protein concentration of the tissue lysate was measured using a Bradford assay (Bio-Rad, Saint Louis, MO, USA) and it was adjusted to 2 mg/mL. Then, the samples were denatured with 2x Laemmli sample buffer (Sigma-Aldrich, Saint Louis, MO, USA) at 100 °C for 10 min and loaded (35 µg) and separated by 10% SDS-PAGE according to molecular weight of the proteins. The proteins in the SDS-PAGE were transferred onto an Immobilon-P polyvinylidene difluoride (PVDF) membrane (Millipore, Billerica, MA, USA) for 2 h using a Trans-Blot semidry transfer cell (Bio-Rad, Hercules, CA, USA) following manufacturers’ standard protein transfer instructions. For immunodetection, the membranes were blocked with Tris-buffered saline containing 5% bovine serum albumin (BSA) and 0.05% Tween20 (TBST) for 1 h, and then probed with target primary antibodies (anti-alkaline phosphatase (intestine) (GeneTex; GTX112100), anti-β-actin (C4) (Santa Cruz Biotechnology; SC-47778)) overnight at 4 °C. After overnight incubation, membranes were washed thrice with PBS containing 0.05% Tween20 (PBST) for 10 min. Then, the membranes were incubated with 1:3000 of horseradish peroxidase (HRP)-conjugated secondary antibody (mouse IgG antibody (HRP) (GeneTex; GTX213111-01) in TBST for 1 h at room temperature. Then, the washing step was repeated thrice (10 min at a time). For the chemiluminescent detection, HRP on the membrane was developed by adding Western blotting detecting reagent (Western Femto ECL Kit, LPS Solution, Daejeon, Republic of Korea) and visualized using chemiluminescence detection system (Fusion Solo S, Vilber, Lourmat, France). At the same time, bands of the IAP and β-actin expression were quantified using Evolution-CAPT software (FUSION software user and service manual v17.03) and normalized with the β-actin to obtain the relative protein expression fold values as compared with control samples.

### 4.7. Histological Analysis

Histological analysis of the jejunum was performed to assess the effects of supplements on gut histomorphometry. Tissues were processed (Leica^®^TP1020 Semi-enclosed Bench-top Tissue Processor, Nussloch, Germany), paraffin-embedded (Leica^®^EG1150 Tissue Embedding Centre, Nussloch, Germany), and 4 μm sections were prepared (Leica^®^RM2125 microtome, Nussloch, Germany). AB (pH 2.5)-PAS staining were carried out under standard protocol to stain both acidic and neutral goblet cells. The sections were observed under light microscope (Leica^®^3000 LED, Wetzlar, Germany), and photographed (Leica DFC450 C, Wetzlar, Germany) at 200× magnification. The images were analyzed using ImageJ 1.5v software. AB-PAS positive goblet cell count, and density, as well as villi height were determined [46].

### 4.8. Statistical Analysis

All data were analyzed for the significant difference (*p* ≤ 0.05) by ANOVA and graphs were drawn using GraphPad Prism software version 5 (GraphPad Software Inc., La Jolla, CA, USA). PCA analysis and PERMANOVA (999 permutations and Bray–Curtis distance) analysis of gut microbiome (relative abundance of families) were performed using VEGAN package of R statistical software ver.3.6.1. Data were shown as means ± standard errors for replicates.

## Figures and Tables

**Table 1:** Comparison of the effect of *S. maxima*-derived modified pectin (SmP) and pectin nanoparticles (SmPNPs) on growth parameters and blood glucose levels in mice.

Parameters	Control	Modified SmP	Modified SmPNPs
Initial BW (g)	20.33 (0.15)	20.70 (0.72)	20.18 (0.79)
Final BW (g)	23.69^a^ (0.17)	23.91^a^ (0.51)	24.53^a^ (0.53)
Water intake (mL/mouse/day)	4.05 (0.07)	4.33* (0.07)	4.34* (0.07)
Total pectin intake (g/4 weeks)	0	45.36	45.64
Weight gain (g/4 weeks)	3.36 (0.12)	3.21 (0.12)	4.35 (0.44)
Weight gain (%/4 weeks)	16.52 (0.7)	15.54 (0.9)	21.65 (2.5)
SGR% (% BW gain/day)	0.55 (0.02)	0.51 (0.03)	0.70 (0.07)
Blood glucose level (mg/dL)	171.25 (19.00)	158.72 (20.00)	165.82 (21.00)
Fasting blood glucose level (mg/mL) at 4th week	145.75 (14.00)	131.83 (26.00)	156.91 (2.00)

^a^ Significantly higher (*p* < 0.05) final weights as compared with initial weight. * Significantly higher (*p* < 0.05) water intake as compared with the control. Standard error of the mean (SEM) is shown in bracket.

**Table 2:** Notable changes in species composition of fecal microbiota and possible effects in the mice gut.

Species of bacteria	Modified SmP vs. control	Modified SmPNPs vs. control	Possible effects	Reference
*Bacteroides acidifaciens*	Decrease	Increase	Prevents obesity. Improves insulin sensitivity.	[27]
*Bacteroides vulgatus*	Decrease	Increase	Reduce gut microbial lipopolysaccharide production. Inhibit Atherosclerosis.	[28]
*Lactobacillus animalis*	Decrease	Increase	Act as probiotic. Reduce chronic inflammation caused by *Mycobacterium avium*.	[29]
*Akkermansia muciniphila*	Decrease	Increase	Adheres to enterocytes. Strengthens the integrity of the epithelial cell layer.	[30]
*Helicobacter hepaticus*	Decrease	Decrease	Cause *H. hepaticus*-induced colitis. Triggers intestinal inflammation.	[31]
*Prevotella loescheii*	NA	Increase	Cause sepsis and soft tissue infections.	[32]

NA, data not available.

## Supplementary Materials

The following are available online at https://www.mdpi.com/1660-3397/18/3/175/s1, Figure S1: Diet-specific changes on taxonomic composition of fecal microbial community of control, modified SmP and SmPNPs treated mice; Figure S2. Graphical representation of PERMANOVA analysis of relative abundance of gut microbiota families in control, modified SmP and SmPNPs, computed by Vegan package of R 3.6.1. with 999 permutations for all comparisons; Table S1: Particle size and zeta potential of modified SmP and modified SmPNPs.; Table S2: Description of the selected genes, related functions and specific primer in this study.
