# Supplementary material for: Marine Microalgae, Spirulina maxima-Derived Modified Pectin and Modified Pectin Nanoparticles Modulate the Gut Microbiota and Trigger Immune Responses in Mice
Source: Mar Drugs. 2020 Mar 21;18(3):175. doi: 10.3390/md18030175 (PMC7143556; doi:10.3390/md18030175)
Supplement: Supplementary file 1 [file marinedrugs-18-00175-s001.pdf]

Supplementary tables

Supplementary table S1. Particle size and zeta potential of modified SmP and modified SmPNPs.

**Product**	**Average particle size (nm)**	**Zeta potential (mV)**
Modified SmP	152.90	-24.4
Modified SmPNPs	64.11	-24.6

Supplementary table S2. Description of the selected genes, related functions and specific primer in this study.

Accession number	Gene nam	Main functions	Primer sequences	
			Forward (5´-3´)	Reverse (5´-3´)
**BC034197.1**	Mucin 2 (Muc2)	Antimicrobial activity	ATGCCCACCTCCTCAAAGAC	GTAGTTTCCGTTGGAACAGTGAAT
NM_023566.3	Mucin 3 (Muc3)	Antimicrobial activity	GTAGTTTCCGTTGGAACAGTGAAT	GCCGTGAATTGTATGAACGGA
NP_034974.1	Mucin 5ac (Muc5ac)	Antimicrobial function	GTGGTTTGACACTGACTTCCC	CTCCTCTCGGTGACAGAGTCT
NM_011575.2	Trefoil factor 3 (Tff3)	Intestinal barrier function	CCTGGTTGCTGGGTCCTCTG	GCCACGGTTGTTACACTGCTC
NR_003146.1	Defensin α pseudogene 1 (Defa-ps1)	Antimicrobial activity	AGACACTAGTCCTCCTCTCTG	CTGGCTGCTCCTCAGTATTAG
NM_183253.3	Defensin α 21 (Def α 21)	Antimicrobial activity	CCAGGGGAAGATGACCAGGCTG	CCAGGGGAAGATGACCAGGCTG
NM_007844.2	Defensin α 29 (Defa29)	Antimicrobial activity	TCCAGGCTGATCCTATCCAA	GCCTCCAAAGGAGACAGAAA
NM_013590.4	Lysozyme 1 (Lyz1)	Antimicrobial function	GAAGCACCGACTATGGGATATT	GATCCCACAGGCATTCTTAGAT
NM_011259.1	Regenerating islet-derived 3α(Reg3 α)	Bacteriocidic mechanisms	CTCCTGCCTGTTGTTTGTATTTC	CATAGCAGTGGGAGCGATAAG
NM_011036.1	Regenerating islet-derived 3 β(Reg3b)	Bacteriocidic mechanisms	TACTGCCTTAGACCGTGCTTTCTG	GACATAGGGCAACTTCACCTCACA
NM_010846.1	Myxovirus resistance protein (Mx1)	Antiviral activity	ACAAGCACAGGAAACCGTATCAG	AGGCAGTTTGGACCATCTTAGTG
NM_015783.3	Interferon stimulated gene 15 (Isg15)	Antiviral activity	CAATGGCCTGGGACCTAAA	CTTCTTCAGTTCTGACACCGTCAT
NM_010502.2	Interferon α 1 (Ifnα 1)	Antiviral activity	CTCTCCTGCCTGAAGGACAGGAAG	GGTGGAGGTCATTGCAGAATGAGT
NM_031168.2	Interleukin 6 (Il6)	Antiinflammatory activity	TCCATCCAGTTGCCTTCTTGG-3	CCACGATTTCCCAGAGAACATG

NM_010548.2	Interleukin 10 (Il10)	Antiinflammatory activity	TTGAATTCCCTGGGTGAGAAG	TCCACTGCCTTGCTCTTATTT
NM_001081082.2	Alkaline phosphatase (Alpi)	Maintain microbiome homeostasis	GGCTACACACTTAGGGGGACCTCCA	AGCTTCGGTGACATTGGGCCGGTT
NM_007432.2	Alkaline phosphatase 3 (Akp3)	Maintain microbiome homeostasis	ACATTGCTACACAACTCATCTCC	TCCTGCCATCCAATCTGGTTC
NM_011577.2	Transforming growth factor β (Tgf-β)	Mediator in gut immunogenic responses	CACCGGAGAGCCCTGGATA	TGTACAGCTGCCGCACACA
NM_010851.3	Myeloid differentiation response gene 88 (Myd88)	Adaptor function in immune mechanisms	TCGATGCCTTTATCTGCTACTG	GGTCGGACACACACAACTTA

Supplementary figures

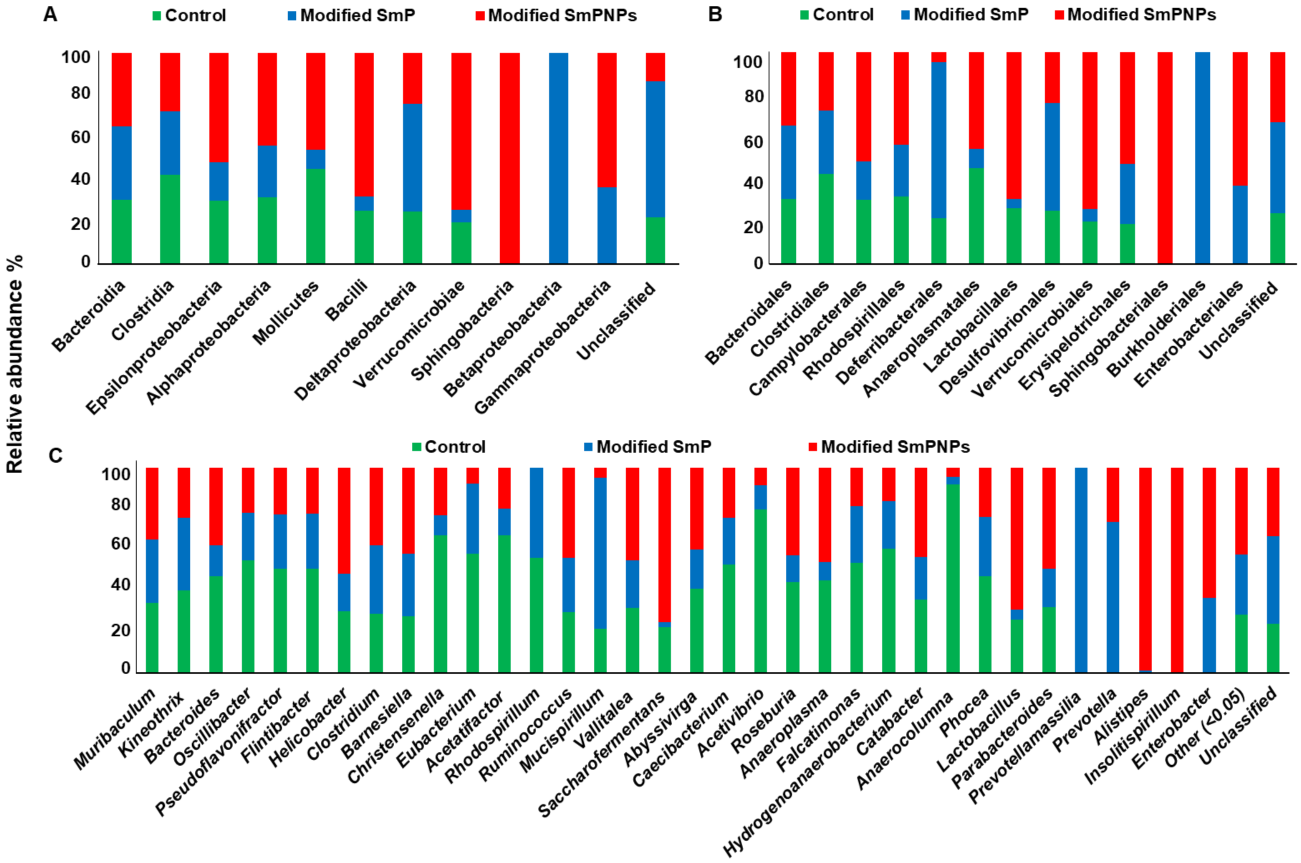

Supplementary figure S1. Diet-specific changes on taxonomic composition of fecal microbial community of control, modified SmP and SmPNPs treated mice; (A) Comparison of order level; (B) Comparison of class level; (C) Comparison of genus level.

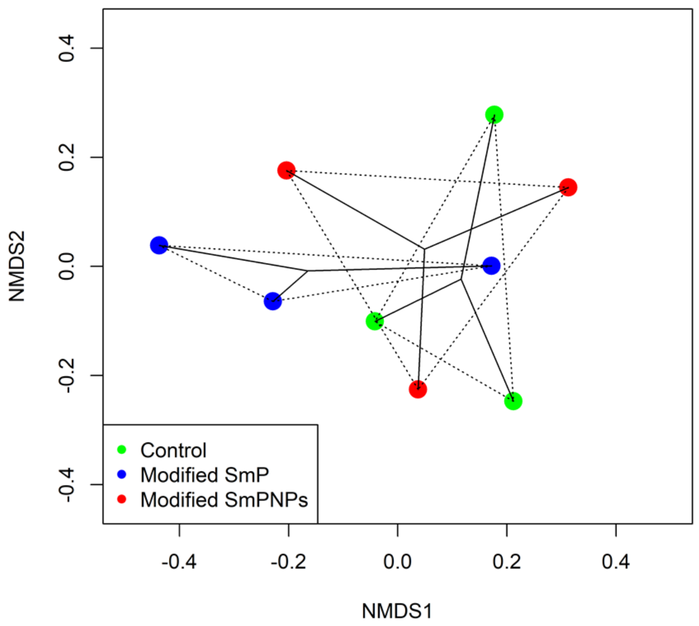

Supplementary figure S2. Graphical representation of PERMANOVA analysis of relative abundance of gut microbiota families in control, modified SmP and SmPNPs, computed by Vegan package of R 3.6.1. with 999 permutations for all comparisons. There were no statistically significant ($p > 0.05$) differences among three groups analyzed. The Bray-Curtis distance matrices are visualized using a nonmetric multiple dimensional scaling ((NMDS1) and NMDS2) plot.
